# Tri- and Difluoromethylated Spiro[5.5]trienones Inhibit the Growth of Cancer Cells In Vitro and In Vivo

**DOI:** 10.3390/biomedicines14040774

**Published:** 2026-03-29

**Authors:** Zhong-Bao Shao, Xiao-Peng Song, Ying-Ying Wang, Yi-Yao Shan, Yu-Meng Xiong, Ke He, Yan Zhang, Zhi Shi

**Affiliations:** 1 Cancer Minimally Invasive Therapies Centre, Guangdong Second Provincial General Hospital, Department of Cell Biology & Institute of Biomedicine, Guangdong Provincial Biotechnology & Engineering Technology Research Center, Guangdong Provincial Key Laboratory of Bioengineering Medicine, Guangdong Basic Research Center of Excellence for Natural Bioactive Molecules and Discovery of Innovative Drugs, Genomic Medicine Engineering Research Center of Ministry of Education, MOE Key Laboratory of Tumor Molecular Biology, National Engineering Research Center of Genetic Medicine, State Key Laboratory of Bioactive Molecules and Druggability Assessment, College of Life Science and Technology, Jinan University, Guangzhou 510632, China; shaozb@stu2022.jnu.edu.cn (Z.-B.S.); songxp@stu2023.jnu.edu.cn (X.-P.S.); wangyingying18@stu2021.jnu.edu.cn (Y.-Y.W.); yiyaoshan@stu2021.jnu.edu.cn (Y.-Y.S.); ymzxx@stu2022.jnu.edu.cn (Y.-M.X.); heke8@mail3.sysu.edu.cn (K.H.); 2 Key Laboratory of the Ministry of Education for Advanced Catalysis Materials, Zhejiang Normal University, Jinhua 321004, China

**Keywords:** trienone, cancer, cell cycle, apoptosis, reactive oxygen species

## Abstract

**Background:** Cancer has emerged as the primary cause of death worldwide in recent years. Current cancer treatment strategies require improvement, creating a pressing need for the development of novel therapeutic agents. This study investigated the anticancer effects of a series of newly synthesized tri- and difluoromethylated spiro[5.5]trienone compounds and evaluated the antitumor efficacy of a lead compound, **3s**. **Methods:** The methyl thiazolyl tetrazolium (MTT) assay was used to assess the effect of the trienone compounds on the growth of cancer cells. Cell cycle distribution and intracellular reactive oxygen species (ROS) levels were analyzed by flow cytometry. Protein expression was examined by Western blot. A mouse xenograft model was utilized to test the anticancer effects and toxicity of **3s** in vivo. **Results:** All 21 tri- and difluoromethylated spiro[5.5]trienones exhibited inhibitory effects on the growth of cancer cells. Among them, compound **3s** showed the strongest inhibitory effect. It induced cell cycle arrest at the G2/M phase and promoted apoptosis. Mechanistically, **3s** activated JNK and ERK signaling and elevated intracellular ROS levels. Furthermore, in a mouse xenograft model, **3s** significantly inhibited tumor growth with minimal toxicity. **Conclusions:** Compound **3s** exhibits potent anticancer efficacy both in vitro and in vivo. The discovery of **3s** offers new potential for cancer therapy.

## 1. Introduction

Cancer is one of the primary causes of human mortality worldwide [1]. According to the latest cancer statistics from 2022, cancer as a major global public health issue imposes a significant societal burden due to its increasing prevalence and high mortality rates [2]. Beyond conventional methods like surgery, chemotherapy, and radiotherapy, advanced modalities including gene therapy [3], immunotherapy [4], photodynamic therapy [5] and monoclonal antibody therapy [6] are increasingly being used. These advanced therapies offer more options for cancer treatment, and some drugs are already in clinical use, but most are still in trials [7,8,9]. Therefore, it is crucial to explore alternative pharmaceutical agents that are both more effective and safer for cancer treatment.

Colorectal cancer is a highly prevalent malignancy. Its development is closely linked to genetic mutations [10] and environmental factors [11]. Recent studies have also identified that lifestyle habits [12], unhealthy diet [13] and lack of exercise [14] are important modifiable risk factors for this disease. Additionally, there is a rising incidence among young adults [15]. Current colorectal cancer treatments encompass surgery, chemotherapy, radiotherapy and immunotherapy [16,17]. Surgery is mainly recommended for early-stage patients, but postoperative complications such as infection, thrombosis, intestinal obstruction, anastomotic leak, and adhesion remain significant concerns [18,19]. In recent years, immunotherapy has gained prominence as a promising therapeutic strategy and also one of the rapidly evolving therapeutic approaches in colorectal cancer treatment [17]. However, its effectiveness is often limited by compensatory inhibitory mechanisms in the antitumor immune response, resulting in acquired drug resistance [20]. Additionally, short follow-up periods hinder the assessment of long-term benefits and adverse events, and comprehensive randomized controlled trials are lacking [21]. Chemotherapeutic regimens for colorectal cancer primarily consist of fluorouracil, irinotecan, capecitabine, and oxaliplatin [22,23]. Moreover, drug resistance limits the therapeutic efficacy, reduces patient survival rates, and ultimately leads to disease recurrence and death [24]. Consequently, there is a pressing need to discover new and effective drugs for colorectal cancer treatment.

Breast cancer is the most common cancer among women, accounting for about one third of all malignancies in women [25,26]. Its global incidence is influenced by an intricate interplay of environmental, genetic, and lifestyle factors, with more than two million new cases diagnosed globally each year [27]. The development of breast cancer is a multifaceted process marked by the accumulation of genetic mutations and various risk factors, such as genetic predisposition, hormonal influences, and unhealthy lifestyle choices [28]. The intricate pathogenesis and varied clinical manifestations of breast cancer pose significant challenges to advancing effective treatment and prevention strategies [29]. Currently, the treatment of early breast cancer mainly includes surgery, hormonal therapy, chemotherapy, radiation and targeted therapy [30]. Despite significant advancements in surgical techniques, controversies in its management persist [31]. Additionally, postoperative complications, such as hematoma and infection, remain significant challenges [32]. Systemic treatment of breast cancer includes chemotherapy (anthracyclines, taxanes, platinums, capecitabine), anti-HER2 therapy (trastuzumab, pertuzumab, trastuzumab emtansine, neratinib), and endocrine therapy (tamoxifen, aromatase inhibitors), among others [33]. Radiotherapy is typically administered after breast-conserving surgery and mastectomy [34]. Combination therapies such as surgery plus drug treatment are the commonly used in the radical treatment of breast cancer [35]. However, clinical outcomes are often suboptimal [36]. This situation highlights the urgent need to discover novel therapeutic agents to improve breast cancer treatment.

Trienone compounds are organic molecules characterized by the presence of three double bonds and a ketone functional group, which constitute an important structural motif widely found in natural products and bioactive pharmaceutical ingredients [37]. In recent years, the biological activities and potential medicinal values of trienone compounds have been increasingly recognized [38]. Ramirez et al. discovered that a new trienone pregnane derivative exhibited potent antiandrogenic effects [39]. The trienonecompound HMPA exhibited superior anti-inflammatory activity to curcumin in RAW264.7 cells [40]. Nuamsee et al. found that trienone analogs of curcuminoids could increase the synthesis of fetal hemoglobin [41]. Wang et al. isolated several novel undescribed trienone diterpenoids, and some of which displayed notable efficacy against several human cancer cells [42]. Furthermore, three new trienone compounds exhibited more potent anti-oral cancer activity than the anticancer drug ellipticine [43]. In this study, we evaluated the anticancer activities of a series of tri- and difluoromethylated spiro[5.5]trienones [44], and identified a lead compound named **3s**, which shows strong anticancer activity both in vitro and in vivo.

## 2. Materials and Methods

### 2.1. Cell Culture and Reagents

Human colorectal cancer cell lines DLD-1, HCT-8, HCT116, HT29, RKO, S1 (from Dr. Zhe-Sheng Chen at St John’s university, New York, NY, USA), SW480, and SW620, human breast cancer cell lines BT474, MCF7, MDA-MB-231, MDA-MB-453, MDA-MB-468, SK-BR-3, and T47D, human lung cancer cell lines H460 and PC9, human liver cancer cell lines MHCC97H and Huh7, human cervical cancer cell line HeLa, human prostate cancer cell line RM1, human osteosarcoma cell line U2OS, mouse colorectal cancer cell line CT26, and mouse breast cancer cell lines 4T1 and C127 were obtained from the Cell Bank of the Chinese Academy of Sciences or as indicated. Cells were cultured in high-glucose Dulbecco’s modified Eagle medium with 10% fetal bovine serum (SA301.02.V, Cellmax, Minhai Company, Lanzhou, China) at 37 °C with 5% CO_2_ in a humidified incubator as previously described [45]. Anti-PARP (9542S), SAPK/JNK (9252S), p-SAPK/JNK (T183/Y185) (4668S), Erk1/2 (4695S), and p-Erk1/2 (T202/Y204) (4370S) antibodies were purchased from Cell Signaling Technology (Boston, MA, USA). Anti-Vinculin antibody (sc-73614) was purchased from Santa Cruz Biotechnology (Santa Cruz, CA, USA). N-acetylcysteine (NAC) (A601127), propidium iodide (PI) (A601112), and skimmed milk (A600669) were ordered from Sangon Biotech (Shanghai, China)

### 2.2. Methyl Thiazolyl Tetrazolium (MTT) Assay

Cells were seeded at 3 × 10^3^ cells/well in 96-well plates (TCP010096, Jet Biofil, Guangzhou, China). Following treatment with the indicated compounds for 72 h, MTT (Q108115, D&B, Shanghai, China) was added to a final concentration of 0.5 mg/mL. After 4 h of incubation, the supernatant was removed, and formazan crystals were dissolved in 50 μL of dimethyl sulfoxide. Absorbance was measured at 570 nm using a BioTek Synergy H1 plate reader (Winooski, VT, USA).

### 2.3. Cell Cycle Assay

Cells were seeded at 1 × 10^5^ cells/well in 6-well plates (TCP010006, Jet Biofil, Guangzhou, China). Following treatment with the indicated compounds for 48 h, cells were fixed with pre-cooled 70% ethanol for 20 min, incubated in staining buffer (50 μg/mL propidium iodide, 0.1% Triton X-100, 0.1% sodium citrate, and 100 μg/mL RNase A) at room temperature in the dark for 20 min, and analyzed using a Beckman Coulter CytoFLEX flow cytometry (Beckman, Brea, CA, USA) at excitation/emission wavelength of 535/617 nm. Data were analyzed by ModFit LT software (version 5.0).

### 2.4. Measurement of Reactive Oxygen Species (ROS)

Cells were treated with **3s** for 48 h and then incubated with 5 μM dihydroethidium (DHE) (M064541, MREDA, Beijing, China) for 30 min, then visualized using an Olympus CKX53SFC microscope (Tokyo, Japan) and analyzed by flow cytometryat excitation/emission wavelength of 535/610 nm. Data were analyzed by FlowJo software (version 10.8).

### 2.5. Western Blot Analysis

Cells were washed twice with pre-cooled PBS and suspended in RIPA lysis buffer (1% NP-40, 1 μM sodium orthovanadate, 0.5% sodium deoxycholate, 0.1% SDS, 0.03% aprotinin,10 ng/mL PMSF) at 4 °C for 30 min. After lysates were centrifuged at 14,000× *g* for 10 min, supernatants were harvested and stored at −80 °C. Protein concentrations were determined with the Bradford assay. Proteins were isolated with 10% sodium dodecyl sulfate polyacrylamide gel electrophoresis and transferred onto polyvinylidene fluoride membranes (IPVH00010, Milipore, Billerica, MA, USA). Membranes were blocked with 5% skimmed milk for 1 h, incubated with primary antibodies for 2 h, followed by horseradish peroxidase-conjugated secondary antibodies for 1 h. After developing with the enhanced chemiluminescence (JXE0011, Jingxin, Guangzhou, China), signals were detected with a ChemiDoc XRS chemiluminescence imaging system (Analytik Jena, Jena, Germany).

### 2.6. MouseXenograft Tumor Assay

Four-week-old female BALB/c mice were purchased from Guangdong Yaokang Technology (Foshan, China) and maintained in a pathogen-free environment. To establish a tumor-bearing mouse model, CT26 cells were injected subcutaneously into the shoulder of mice. Mice were randomly divided into four groups: control, oxaliplatin 5 mg/kg, **3s** (5 and 15 mg/kg). Once every two days, mice received injections of oxaliplatinor compound **3s** via i.p. injection. The tumor volume and the weight of mice were measured every two days. Tumor volume was calculated using the formula: V = $\frac{\pi }{6}\times {\left(\frac{X+Y}{2}\right)}^{3}$. ‘*X*’ and ‘*Y*’ represent the long and short diameter of the tumors, respectively. At the experimental endpoint, blood was collected, and mice were euthanized. All procedures were approved by the Laboratory Animal Center of Jinan University.

### 2.7. Measurement of Serum Alanine Transaminase (ALT), Aspartate Transaminase (AST), Creatinine (Cr), and Urea Nitrogen (BUN)

Serum samples were separated from blood of mice by centrifugation at 4 °C (3000 rpm, 30 min). The serum ALT, AST, Cr, and BUN were detected as described by the commercial kits (C010-2-1, C009-2-1, C011-2-1, C013-2-1, Jiancheng, Nanjing, China). To detect the serum ALT and AST levels, 5 μL of test samples and 20 μL of reagent A were mixed and incubated at 37 °C for 30 min. A total of 20 μL of reagent B was added and incubated at 37 °C for 20 min. Then, 20 μL of reagent C was added and incubated at room temperature for 15 min. Finally, the absorbance values were measured at the wavelength of 505 nm. The corresponding ALT/AST activity values were obtained by substituting the values into the standard curve. To detect the serum Cr level, 6 μL of test samples and 180 μL of reagent A were mixed and incubated at 37 °C for 5 min. Subsequently, the absorbance values (A1) were measured at the wavelength of 546 nm. After that, 20 μL of reagent B was added and incubated at room temperature for 5 min. Finally, the absorbance values (A2) were measured at the wavelength of 546 nm. The calculation formula is as follows: CRE levels (μM) = $\frac{{∆A}_{test}-{∆A}_{blank}}{{∆A}_{standard}-{∆A}_{blank}}\times C_{standard}$, ∆A = A2 − K × A1. K is the dilution factor and $C_{standard}$ = 442 μM. To detect the serum BUN level, 200 μL of test samples and 250 μL of reagent A were mixed and incubated at 37 °C for 5 min. After that, 1 mL each of reagents B and C were added and incubated at 37 °C for 10 min. At last, the absorbance values (A) were measured at the wavelength of 640 nm. BUN (mM) = $\frac{A_{test}-A_{blank}}{A_{standard}-A_{blank}}\times C_{standard}$ × N. *C_standard_* = 10 mM, N represents the dilution factor.

### 2.8. Statistical Analysis

Statistical significance was determined using Student’s *t*-test in GraphPad Prism 9. *p* < 0.05 was considered statistically significant.

## 3. Results

### 3.1. Tri- and Difluoromethylated Spiro[5.5]trienones Inhibit the Growth of Cancer Cells In Vitro

**Figure 1:**
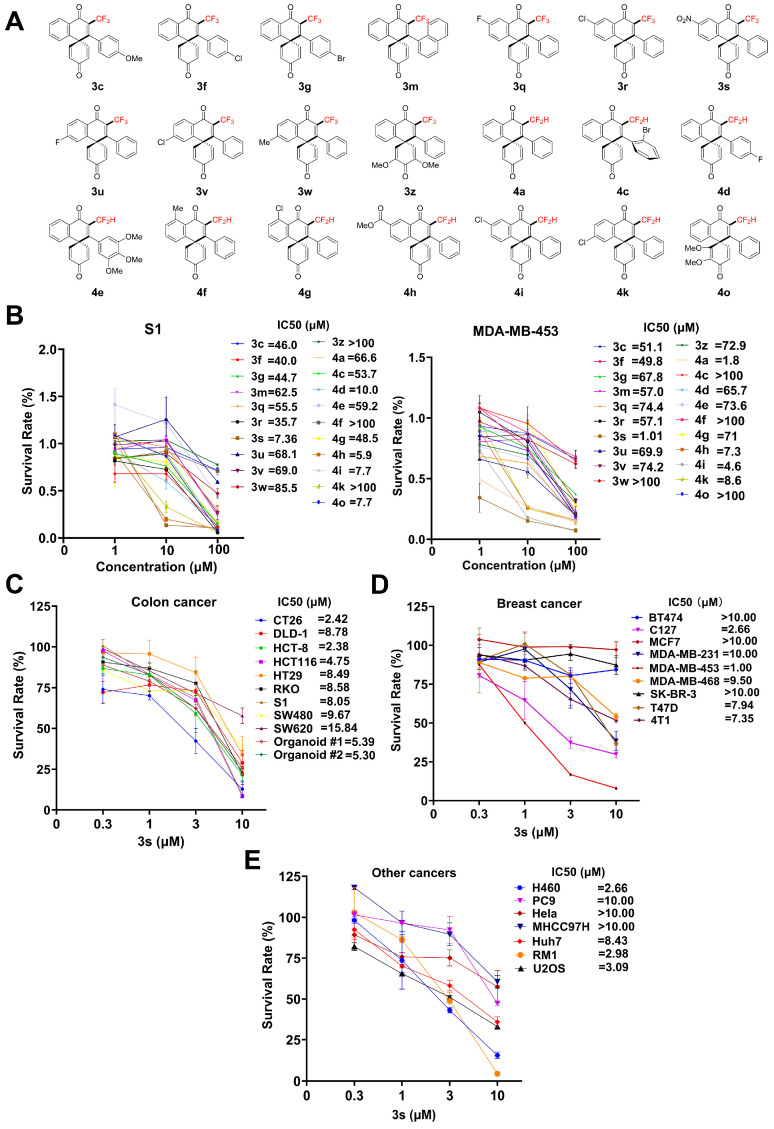
Tri- and difluoromethylated spiro[5.5]trienones inhibit the growth of cancer cells in vitro. (**A**) Chemical structures. (**B**) Survival rates and IC_50_ values of S1 and MDA-MB-453 cells treated with different compounds (1, 10, 100 μM) for 72 h. (**C**–**E**) Survival rates and IC_50_ values of (**C**) colorectal, (**D**) breast, and (**E**) other cancer cell lines treated with compound **3s**. Data are presented as mean ± standard deviation (SD) (n = 3).

The chemical structures of 21 tri- and difluoromethylated spiro[5.5]trienones are shown in Figure 1A. Their effects on the proliferation of human colorectal cancer S1 and breast cancer MDA-MB-453 cells were first evaluated using the MTT assay. As shown in Figure 1B, all compounds inhibited cell growth. Analysis of the structure–activity relationship indicates compounds **3s** and **4h** showed the effective inhibition in both S1 and MDA-MB-453 cancer cells, possibly due to their special nitro or methoxy group. Compound **3s** exhibited the most potent effect and was selected for further study. Its efficacy was then confirmed in a broader panel of cancer cell lines, showing particularly strong activity against CT26 and MDA-MB-453 cells (Figure 1C–E).

### 3.2. ***3s*** Induces Cell Cycle Arrest at the G2/M Phase and Apoptosis

**Figure 2:**
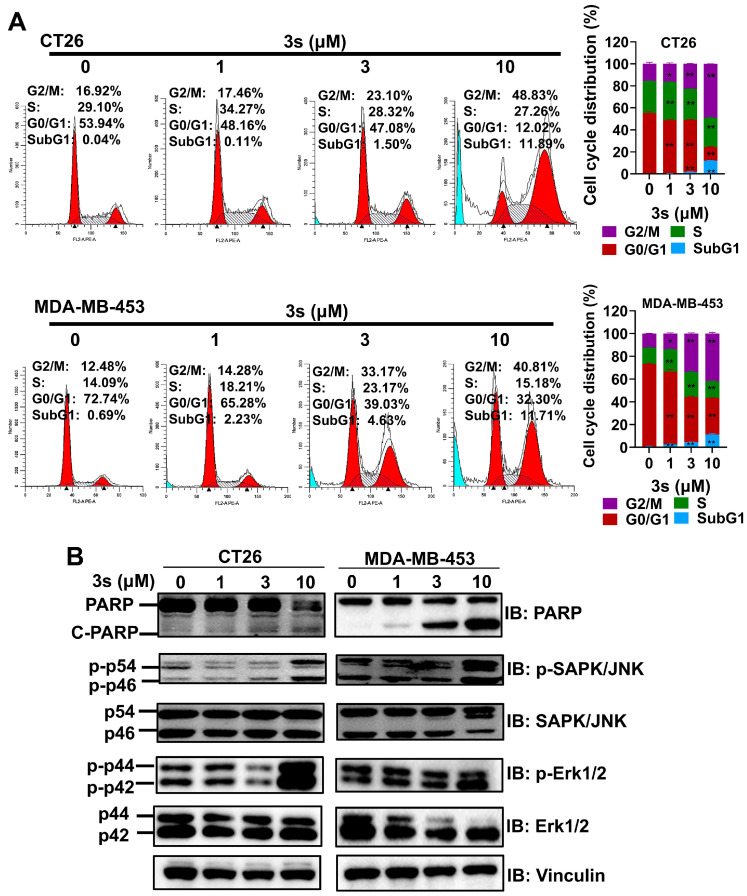
**3s** induces cell cycle arrest at the G2/M phase and apoptosis. (**A**) Cell cycle analysis of CT26 and MDA-MB-453 cells treated with **3s** for 48 h. (**B**) Western blot analysis of PARP cleavage, and JNK/ERK phosphorylation. Data are mean ± SD (n = 3). * *p* < 0.05, ** *p* < 0.01 vs. control.

To determine the mechanism, cell cycle distribution was analyzed. Treatment with **3s** (1, 3, 10 μM) for 48 h caused a dose-dependent increase in the proportion of cells in the G2/M and sub-G1 (apoptotic) phases in both CT26 and MDA-MB-453 cells (Figure 2A). Western blot analysis confirmed increased levels of cleaved PARP, as well as phosphorylated JNK and ERK (Figure 2B). These data suggest that **3s** induces cell cycle arrest at the G2/M phase and apoptosis.

### 3.3. ***3s*** Elevates Intracellular ROS Levels

**Figure 3:**
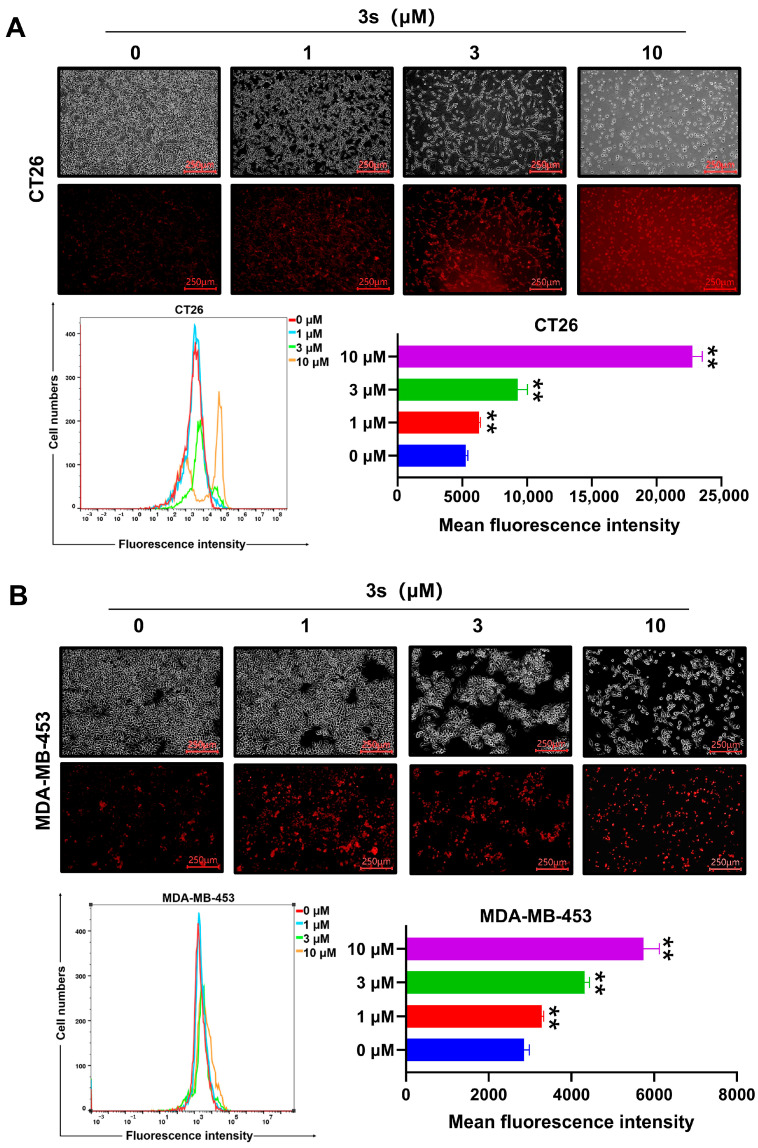
**3s** elevates intracellular ROS levels. CT26 (**A**) and MDA-MB-453 (**B**) cells were treated with 3s for 48 h and stained with DHE. Representative images and quantitative flow cytometry data are shown. Data are presented as mean ± SD (n = 3). ** *p* < 0.01 vs. control.

ROS plays a critical role in the cell death induced by many anticancer agents. We next examined oxidative stress. Cells treated with **3s** showed a dose-dependent increase in intracellular ROS levels, as detected by DHE staining, concomitant with reduced cell viability (Figure 3A,B).

### 3.4. NAC Reverses ***3s***-Induced ROS Elevation and Growth Inhibition

**Figure 4:**
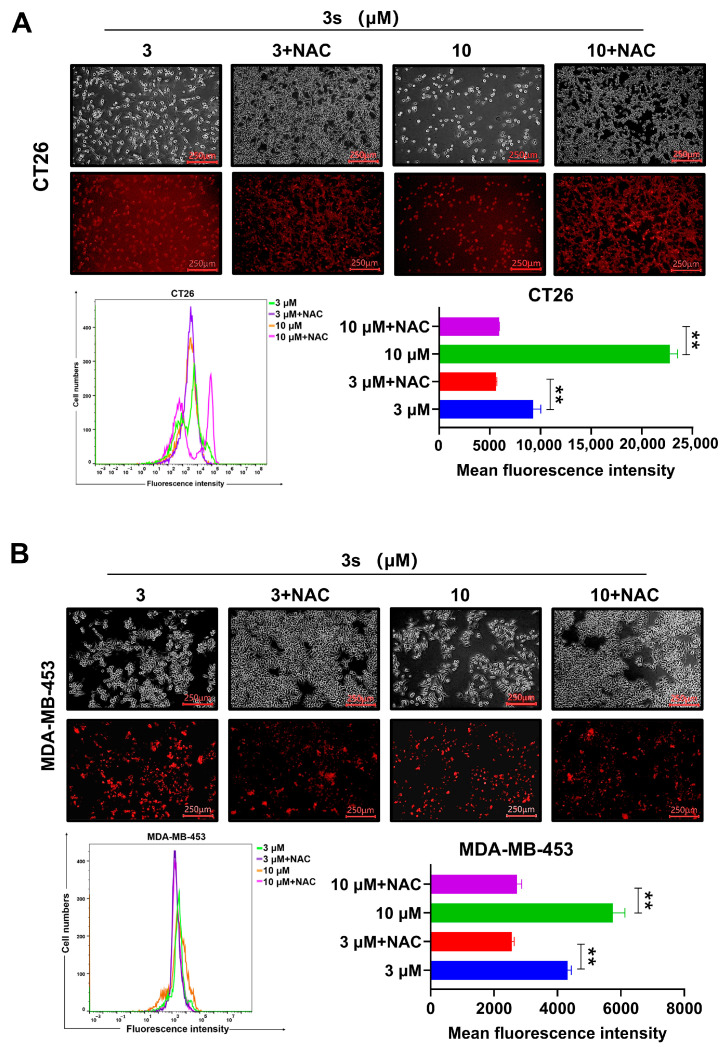
NAC reverses **3s**-induced ROS generation and growth inhibition. CT26 (**A**) and MDA-MB-453 (**B**) cells were pretreated with or without NAC (5 mM) for 1 h before **3s** treatment for 48 h. Intracellular ROS and cell viability were assessed. Data are presented as mean ± SD (n = 3). ** *p* < 0.01 vs. corresponding control.

NAC has been used as a direct scavenger of ROS and an antioxidant in cancer biology [46]. To confirm the role of ROS, cells were pretreated with the antioxidant NAC. NAC pretreatment significantly attenuated the **3s**-induced increase in ROS and rescued cell viability (Figure 4A,B), indicating that ROS generation is crucial for **3s**-mediated growth inhibition.

### 3.5. ***3s*** Inhibits Tumor Growth In Vivo

**Figure 5:**
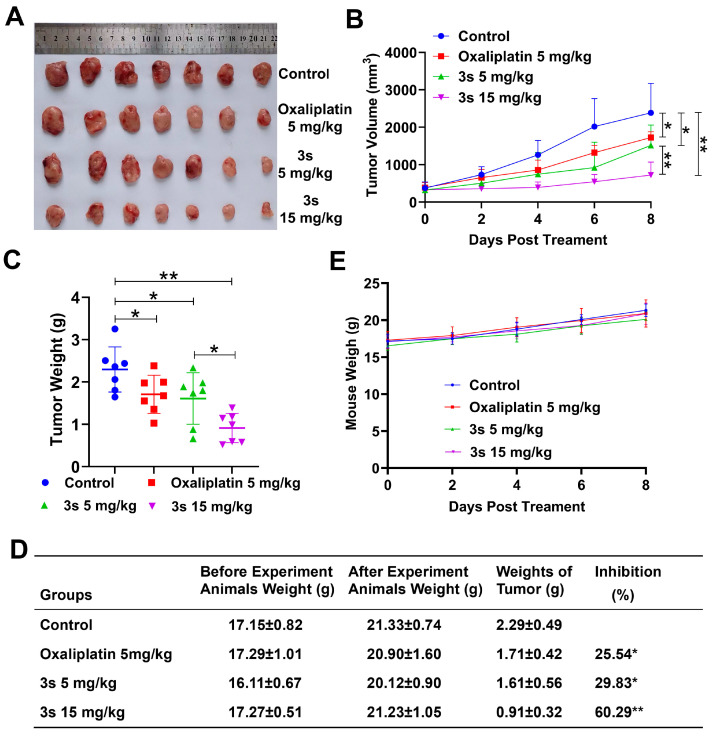
**3s** inhibits tumor growth in vivo. (**A**) Excised tumors. (**B**) Tumor growth curves. (**C**) Final tumor weights. (**D**) Tumor inhibition rates. (**E**) Body weight changes. Data are presented as mean ± SD (n = 7). * *p* < 0.05, ** *p* < 0.01 vs. control.

The antitumor efficacy of **3s** was evaluated in a CT26 xenograft model. **3s** treatment significantly suppressed tumor growth in a dose-dependent manner (Figure 5A–C). At 5 and 15 mg/kg, tumor inhibition rates were 29.83% and 60.29%, respectively, compared to 25.54% for oxaliplatin (5 mg/kg) (Figure 5E). No significant body weight loss was observed (Figure 5D). These results indicate that **3s** inhibits tumor growth in vivo.

### 3.6. In Vivo Toxicity Profile of ***3s***

**Figure 6:**
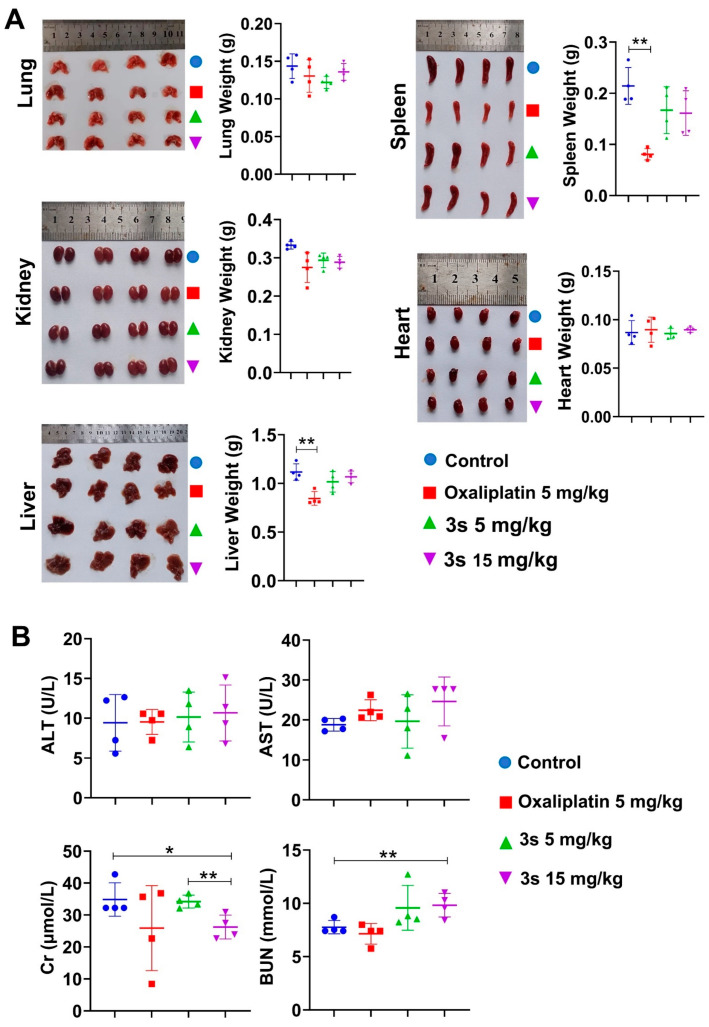
In vivo toxicity profile of 3s. (**A**) Weights of major organs. (**B**) Serum levels of ALT, AST, Cr, and BUN. Data are presented as mean ± SD (n = 4). * *p* < 0.05, ** *p* < 0.01 vs. control.

To assess safety, organ weights and serum biochemistry were analyzed. **3s** did not significantly affect the weights of major organs (Figure 6A), whereas oxaliplatin reduced spleen and liver weights. Serum ALT and AST levels were unchanged by **3s**, though minor alterations in Cr and BUN were noted at the high dose (Figure 6B).

## 4. Discussion

Currently, screening anticancer drugs based on the molecular biological characteristics of tumors is a prevalent strategy in cancer treatment [47]. Research into cell cycle control mechanisms offers promising insights for cancer treatment [48]. Drugs that induce cell cycle arrest and apoptosis hold potential benefits for combating cancer [49,50], while research on the anticancer effects of trienones remains limited. Wang et al. reported a few trienones which demonstrated significant activity against prostate and cervical cells and leading to obvious cell cycle arrest and inducing apoptosis. Notably, some trienones had 5- to 36-fold more potential than curcumin [51]. Munck et al. reported that the IC_50_ value of curcumin in HCT-116 was >40 μM [52], while that of **3s** was 4.75 μM. And Dal et al. reported that the IC_50_ values of curcumin for HCT116, SW480, and HT29 were 19.05, 15.63 and 22.10 μM, respectively [53], while in our study, **3s** was more potent than curcumin in the inhibition of cell viability in those three human colorectal cancer cell lines. In another study, the IC_50_ of curcuminfor MDA-MA-231 cells was 24.7 μM [54], which is higher than the IC_50_ of **3s** (10 μM). Yugandhar et al. synthesized a series of trienones and found that these trienones exhibited potent inhibitory effects against MCF7, DU145, A549, and HepG2 cells. Among them, compounds 9b and 9e were able to induce cell cycle arrest and mitochondria-mediated apoptosis [55]. Our findings are consistent, identifying compound **3s** as a potent inhibitor that induces G2/M arrest and apoptosis.

Elevated ROS levels can promote the development of the cancer phenotype and make cancer cells more susceptible to apoptosis [56,57]. Investigating the mechanisms that regulate ROS levels could provide valuable insights for designing targeted oxidative stress therapies for cancer [58,59]. Utaipan et al. reported that a synthetic trienone analog of curcumin, 1,7-bis(3-hydroxyphenyl)-1,4,6-heptatrien-3-one, induced ROS-mediated cell death, through the activation of caspase 3, 7 and 9 pathways in human oral squamous cells exhibiting multidrug resistance (CLS-354/DX) [60]. Our study confirms that **3s** elevates ROS, and the ROS scavenger NAC reverses its cytotoxicity, supporting a key role for oxidative stress in its mechanism.

Oxaliplatin, a third-generation platinum-based chemotherapy agent, causes fewer and milder adverse reactions than other platinum-based agents [61]. Moreover, oxaliplatin exhibits superior inhibitory effects on colorectal cancer [62], lung cancer [63] and breast cancer cells [64,65]. In our model, **3s** showed comparable or superior efficacy to oxaliplatin without causing significant organ weight loss, suggesting a potentially better safety profile.

The JNK and ERK pathway are important intracellular signaling pathways, involved in cell proliferation and regulating cell apoptosis and other cellular physiological processes [66,67]. The activation of the JNK signaling pathway leads to cell apoptosis in CRC cancer [68] and breast cancer cells [69]. The inhibition of the ERK signaling pathway induces apoptosis in breast cancer [70] and colorectal cancer cells [71]. However, reports on the induction of ERK pathway activation to promote cell apoptosis are also increasing, and the mechanism of the ERK pathway in regulating cell apoptosis remains to be fully elucidated [72]. We observed that **3s** activates both pathways. The concurrent activation of pro-apoptotic JNK and the context-dependent ERK pathway suggests **3s** may engage a multifaceted signaling network to induce cell death.

## 5. Conclusions

In conclusion, compound **3s** potently inhibits cancer cell growth in vitro and in vivo by inducing G2/M arrest, elevating ROS, activating JNK/ERK signaling, and promoting apoptosis. It demonstrates a favorable efficacy and toxicity profile compared to oxaliplatin in our models. The discovery of **3s** offers new potential for cancer therapy.

## Data Availability

The original contributions presented in this study are included in the article. Further inquiries can be directed to the corresponding authors.

## References

[1] Jassim A., Rahrmann E.P., Simons B.D., Gilbertson R.J. (2023). Cancers make their own luck: Theories of cancer origins. Nat. Rev. Cancer.

[2] Bray F., Laversanne M., Sung H., Ferlay J., Siegel R.L., Soerjomataram I., Jemal A. (2024). Global cancer statistics 2022: GLOBOCAN estimates of incidence and mortality worldwide for 36 cancers in 185 countries. CA Cancer J. Clin..

[3] Cesur-Ergün B., Demir-Dora D. (2023). Gene therapy in cancer. J. Gene Med..

[4] Weng Z., Zhang B., Wu C., Yu F., Han B., Li B., Li L. (2021). Therapeutic roles of mesenchymal stem cell-derived extracellular vesicles in cancer. J. Hematol. Oncol..

[5] Ji B., Wei M., Yang B. (2022). Recent advances in nanomedicines for photodynamic therapy (PDT)-driven cancer immunotherapy. Theranostics.

[6] Zinn S., Vazquez-Lombardi R., Zimmermann C., Sapra P., Jermutus L., Christ D. (2023). Advances in antibody-based therapy in oncology. Nat. Cancer.

[7] Dastjerd N.T., Valibeik A., Rahimi Monfared S., Goodarzi G., Moradi Sarabi M., Hajabdollahi F., Maniati M., Amri J., Samavarchi Tehrani S. (2022). Gene therapy: A promising approach for breast cancer treatment. Cell Biochem. Funct..

[8] Delgado M., Garcia-Sanz J.A. (2023). Therapeutic Monoclonal Antibodies against Cancer: Present and Future. Cells.

[9] Lu X., Li Y., Li Y., Zhang X., Shi J., Feng H., Yu Z., Gao Y. (2023). Prognostic and predictive biomarkers for anti-EGFR monoclonal antibody therapy in RAS wild-type metastatic colorectal cancer: A systematic review and meta-analysis. BMC Cancer.

[10] Dosunmu G.T., Shergill A. (2024). Colorectal Cancer: Genetic Underpinning and Molecular Therapeutics for Precision Medicine. Genes.

[11] Murphy C.C., Zaki T.A. (2024). Changing epidemiology of colorectal cancer—birth cohort effects and emerging risk factors. Nat. Rev. Gastroenterol. Hepatol..

[12] Puzzono M., Mannucci A., Di Leo M., Zuppardo R.A., Russo M., Ditonno I., Goni E., Notaristefano C., Azzolini F., Fanti L. (2022). Diet and Lifestyle Habits in Early-Onset Colorectal Cancer: A Pilot Case-Control Study. Dig. Dis..

[13] Vernia F., Longo S., Stefanelli G., Viscido A., Latella G. (2021). Dietary Factors Modulating Colorectal Carcinogenesis. Nutrients.

[14] Rezende L.F.M., Ferrari G., Bahia L.R., Rosa R.D.S., da Rosa M.Q.M., de Souza R.C., Lee D.H., Giovannucci E., Eluf-Neto J. (2021). Economic burden of colorectal and breast cancers attributable to lack of physical activity in Brazil. BMC Public Health.

[15] Spaander M.C.W., Zauber A.G., Syngal S., Blaser M.J., Sung J.J., You Y.N., Kuipers E.J. (2023). Young-onset colorectal cancer. Nat. Rev. Dis. Primers.

[16] Adebayo A.S., Agbaje K., Adesina S.K., Olajubutu O. (2023). Colorectal Cancer: Disease Process, Current Treatment Options, and Future Perspectives. Pharmaceutics.

[17] Fan A., Wang B., Wang X., Nie Y., Fan D., Zhao X., Lu Y. (2021). Immunotherapy in colorectal cancer: Current achievements and future perspective. Int. J. Biol. Sci..

[18] Pallan A., Dedelaite M., Mirajkar N., Newman P.A., Plowright J., Ashraf S. (2021). Postoperative complications of colorectal cancer. Clin. Radiol..

[19] Pak H., Maghsoudi L.H., Soltanian A., Gholami F. (2020). Surgical complications in colorectal cancer patients. Ann. Med. Surg..

[20] Yu B., Kang J., Lei H., Li Z., Yang H., Zhang M. (2024). Immunotherapy for colorectal cancer. Front. Immunol..

[21] Shi J., Sun Z., Gao Z., Huang D., Hong H., Gu J. (2023). Radioimmunotherapy in colorectal cancer treatment: Present and future. Front. Immunol..

[22] Manzi J., Hoff C.O., Ferreira R., Pimentel A., Datta J., Livingstone A.S., Vianna R., Abreu P. (2023). Targeted Therapies in Colorectal Cancer: Recent Advances in Biomarkers, Landmark Trials, and Future Perspectives. Cancers.

[23] Ciardiello F., Ciardiello D., Martini G., Napolitano S., Tabernero J., Cervantes A. (2022). Clinical management of metastatic colorectal cancer in the era of precision medicine. CA Cancer J. Clin..

[24] Kumar A., Gautam V., Sandhu A., Rawat K., Sharma A., Saha L. (2023). Current and emerging therapeutic approaches for colorectal cancer: A comprehensive review. World J. Gastrointest. Surg..

[25] Siegel R.L., Giaquinto A.N., Jemal A. (2024). Cancer statistics, 2024. CA Cancer J. Clin..

[26] Xu Y., Gong M., Wang Y., Yang Y., Liu S., Zeng Q. (2023). Global trends and forecasts of breast cancer incidence and deaths. Sci. Data.

[27] Wilkinson L., Gathani T. (2021). Understanding breast cancer as a global health concern. Br. J. Radiol..

[28] Xiong X., Zheng L.W., Ding Y., Chen Y.F., Cai Y.W., Wang L.P., Huang L., Liu C.C., Shao Z.M., Yu K.D. (2025). Breast cancer: Pathogenesis and treatments. Signal Transduct. Target. Ther..

[29] Giaquinto A.N., Sung H., Miller K.D., Kramer J.L., Newman L.A., Minihan A., Jemal A., Siegel R.L. (2022). Breast Cancer Statistics, 2022. CA Cancer J. Clin..

[30] Ben-Dror J., Shalamov M., Sonnenblick A. (2022). The History of Early Breast Cancer Treatment. Genes.

[31] Gutnik L., Fayanju O.M. (2021). Controversies in Breast Cancer Surgery. Surg. Clin. N. Am..

[32] Al-Hilli Z., Wilkerson A. (2021). Breast Surgery: Management of Postoperative Complications Following Operations for Breast Cancer. Surg. Clin. N. Am..

[33] Sheikh M.S., Ying H. (2023). Antibody-drug Conjugates for Breast Cancer Treatment. Recent Pat. Anti-Cancer Drug Discov..

[34] Kerr A.J., Dodwell D., McGale P., Holt F., Duane F., Mannu G., Darby S.C., Taylor C.W. (2022). Adjuvant and neoadjuvant breast cancer treatments: A systematic review of their effects on mortality. Cancer Treat. Rev..

[35] Khan M.M., Yalamarty S.S.K., Rajmalani B.A., Filipczak N., Torchilin V.P. (2024). Recent strategies to overcome breast cancer resistance. Crit. Rev. Oncol. Hematol..

[36] Tao X., Li T., Gandomkar Z., Brennan P.C., Reed W.M. (2023). Incidence, mortality, survival, and disease burden of breast cancer in China compared to other developed countries. Asia-Pac. J. Clin. Oncol..

[37] Tang J., Luo W.K., Zhou J. (2023). Advances in the Synthesis of Azaspiro[4.5]trienones. Chin. J. Org. Chem..

[38] Zhou X., Wang J., Ma D., Shen Y., Zhao Y., Wu J. (2024). Electrochemical synthesis of phosphorylated azaspiro[4.5]di/trienones through dearomative spirocyclization. Chem. Commun..

[39] Ramirez E., Cabeza M., Heuze I., Gutiérrez E., Bratoeff E., Membrillo M., Lira A. (2002). Synthesis and Pharmacological Evaluation of New 16-Methyl Pregnane Derivatives. Chem. Pharm. Bull..

[40] Jansakun C., Chulrik W., Chaichompoo W., Yotmanee P., Lehboon K., Chunglok W., Sattayakhom A., Hiransai P., Kamdee K., Utaipan T. (2021). 1,7-Bis(4-hydroxy-3-methoxyphenyl)-1,4,6-heptatrien-3-one alleviates lipopolysaccharide-induced inflammation by targeting NF-κB translocation in murine macrophages and it interacts with MD2 *in silico*. Mol. Med. Rep..

[41] Nuamsee K., Chuprajob T., Pabuprapap W., Jintaridth P., Munkongdee T., Phannasil P., Vadolas J., Chaichompoo P., Suksamrarn A., Svasti S. (2021). Trienone analogs of curcuminoids induce fetal hemoglobin synthesis via demethylation at Gγ-globin gene promoter. Sci. Rep..

[42] Wang D.D., Zhang R., Tang L.Y., Wang L.N.Q., Ao M.R., Jia J.M., Wang A.H. (2024). Identification of diterpenoids from Salvia castanea Diels f. tomentosa Stib and their antitumor activities. Bioorganic Chem..

[43] Chuprajob T., Changtam C., Chokchaisiri R., Chunglok W., Sornkaew N., Suksamrarn A. (2014). Synthesis, cytotoxicity against human oral cancer KB cells and structure–activity relationship studies of trienone analogues of curcuminoids. Bioorg. Med. Chem. Lett..

[44] Zhang Y., Ma C., Struwe J., Feng J., Zhu G., Ackermann L. (2021). Electrooxidative dearomatization of biaryls: Synthesis of tri- and difluoromethylated spiro[5.5]trienones. Chem. Sci..

[45] Zhao Z., He J., Qiu S., Wang L., Huangfu S., Hu Y., Wu Q., Yang Y., Li X., Huang M. (2025). Targeting PLK1-CBX8-GPX4 axis overcomes BRAF/EGFR inhibitor resistance in BRAFV600E colorectal cancer via ferroptosis. Nat. Commun..

[46] Kalyanaraman B. (2022). NAC, NAC, Knockin’ on Heaven’s door: Interpreting the mechanism of action of N-acetylcysteine in tumor and immune cells. Redox Biol..

[47] Zheng Y., Zhang X., Su Z. (2021). Design of metal–organic framework composites in anti-cancer therapies. Nanoscale.

[48] Glaviano A., Singh S.K., Lee E.H.C., Okina E., Lam H.Y., Carbone D., Reddy E.P., O’Connor M.J., Koff A., Singh G. (2025). Cell cycle dysregulation in cancer. Pharmacol. Rev..

[49] Tsai T.-H., Lee K.-T., Hsu Y.-C. (2023). JSI-124 Induces Cell Cycle Arrest and Regulates the Apoptosis in Glioblastoma Cells. Biomedicines.

[50] Yin F., Zhao R., Gorja D.R., Fu X., Lu N., Huang H., Xu B., Chen H., Shim J.-H., Liu K. (2022). Novel dual inhibitor for targeting PIM1 and FGFR1 kinases inhibits colorectal cancer growth in vitro and patient-derived xenografts in vivo. Acta Pharm. Sin. B.

[51] Wang R., Zhang X., Chen C., Chen G., Zhong Q., Zhang Q., Zheng S., Wang G., Chen Q.-H. (2016). Synthesis and evaluation of 1,7-diheteroarylhepta-1,4,6-trien-3-ones as curcumin-based anticancer agents. Eur. J. Med. Chem..

[52] De Munck J., Grootaert C., Magdalenic K., Deveci D., Gansemans Y., Van Nieuwerburgh F., Boon N., Skirtach A., Rajkovic A., D’hooghe M. (2026). Curcumin-based benzothiazepane analogues exhibit selective anti-cancer activity in HCT-116 cells via precipitated particle formation and internalisation. Biomed. Pharmacother..

[53] Dal Z., Aru B. (2023). The role of curcumin on apoptosis and NLRP3 inflammasome-dependent pyroptosis on colorectal cancer in vitro. Turk. J. Med. Sci..

[54] Alsaikhan F. (2026). The synergistic effect of resveratrol and curcumin on oncogenic lncRNA MAPT-IT1 and ferroptosis-related genes in breast cancer. Naunyn-Schmiedeberg’s Arch. Pharmacol..

[55] Yugandhar D., Nayak V.L., Archana S., Shekar K.C., Srivastava A.K. (2015). Design, synthesis and anticancer properties of novel oxa/azaspiro[4,5]trienones as potent apoptosis inducers through mitochondrial disruption. Eur. J. Med. Chem..

[56] Cheung E.C., Vousden K.A.-O. (2022). The role of ROS in tumour development and progression. Nat. Rev. Cancer.

[57] Chiu H.-W., Hung S.-W., Chiu C.-F., Hong J.-R. (2023). A Mitochondrion-Targeting Protein (B2) Primes ROS/Nrf2-Mediated Stress Signals, Triggering Apoptosis and Necroptosis in Lung Cancer. Biomedicines.

[58] Glorieux C., Liu S., Trachootham D., Huang P. (2024). Targeting ROS in cancer: Rationale and strategies. Nat. Rev. Drug Discov..

[59] Ahn C.R., Kim H.I., Kim J.-E., Ha I.J., Ahn K.S., Park J., Kim Y.W., Baek S.H. (2023). Ponciri Fructus Immatarus Sensitizes the Apoptotic Effect of Hyperthermia Treatment in AGS Gastric Cancer Cells through ROS-Dependent HSP Suppression. Biomedicines.

[60] Utaipan T., Boonyanuphong P., Chuprajob T., Suksamrarn A., Chunglok W. (2020). A trienone analog of curcumin, 1,7-bis(3-hydroxyphenyl)-1,4,6-heptatrien-3-one, possesses ROS- and caspase-mediated apoptosis in human oral squamous cell carcinoma cells in vitro. Appl. Biol. Chem..

[61] Kang L., Tian Y., Xu S., Chen H. (2021). Oxaliplatin-induced peripheral neuropathy: Clinical features, mechanisms, prevention and treatment. J. Neurol..

[62] Hanie M., Mona M., Zahra M., Amir A., Mohammad Hossein S., Reihaneh Alsadat M., Elnaz G., Gordon A.F., Hamed M., Shaho M. (2023). Nanoparticles Containing Oxaliplatin and the Treatment of Colorectal Cancer. Curr. Pharm. Des..

[63] Ren K., Wang J., Li Y., Li Z., Wu K., Zhou Z., Li Y., Han X. (2022). The Efficacy of Drug-eluting Bead Transarterial Chemoembolization Loaded With Oxaliplatin for the Treatment of Stage III-IV Non-small-cell Lung Cancer. Acad. Radiol..

[64] Andreidesz K., Koszegi B., Kovacs D., Bagone Vantus V., Gallyas F., Kovacs K. (2021). Effect of Oxaliplatin, Olaparib and LY294002 in Combination on Triple-Negative Breast Cancer Cells. Int. J. Mol. Sci..

[65] Ding B., Liu X., Li Z., Xie X., Li J., Wang J., Li S., Wang P., Xie Y., Ma X. (2025). A novel platinum(IV) prodrug, gramine-Pt(IV) enhances chemoimmunotherapy by activating cGAS-STING and modulating TGF-β-MHC-I axis. Drug Resist. Updates.

[66] Jiang M.-Z., Li C., Mao C.-M., Yu H., Zhou Y.-C., Pu S.-Q., Li R.-Z., Liao Y.-J., Zhang D.-Y., Yang P. (2024). The MAPK/ERK signaling pathway involved in Raddeanin A induces apoptosis via the mitochondrial pathway and G2 phase arrest in multiple myeloma. Sci. Rep..

[67] Moon J.-M., Lee S.-W., Jang Y.-S., Lee S.-A., Jung S.-H., Kim S.-K., Park B.-K., Park Y.-S., Kim B.-S., Yang M.-S. (2025). Gossypin induces apoptosis and autophagy via the MAPK/JNK pathway in HT-29 human colorectal cancer cells. Int. J. Mol. Med..

[68] Jiang X., Li G., Zhu B., Zang J., Lan T., Jiang R., Wang B. (2023). p20BAP31 induces cell apoptosis via both AIF caspase-independent and the ROS/JNK mitochondrial pathway in colorectal cancer. Cell. Mol. Biol. Lett..

[69] Zhang Z., Zhang H., Li D., Zhou X., Qin Q., Zhang Q. (2021). Caspase-3-mediated GSDME induced Pyroptosis in breast cancer cells through the ROS/JNK signalling pathway. J. Cell. Mol. Med..

[70] Jiang G., Zhou X., Hu Y., Tan X., Wang D., Yang L., Zhang Q., Liu S. (2024). The antipsychotic drug pimozide promotes apoptosis through the RAF/ERK pathway and enhances autophagy in breast cancer cells. Cancer Biol. Ther..

[71] Peng J., Lu J., Li G.-H., Ma M.-M., Mou Y.-P., Zhu Q.-C. (2025). Curzerene Induces Apoptosis in Colorectal Cancer Cells Through Inhibition of MEK/ERK Signaling Pathway. Chin. J. Integr. Med..

[72] Sugiura R., Satoh R., Takasaki T. (2021). ERK: A Double-Edged Sword in Cancer. ERK-Dependent Apoptosis as a Potential Therapeutic Strategy for Cancer. Cells.

